# An exploration of the gut and environmental resistome in a community in northern Vietnam in relation to antibiotic use

**DOI:** 10.1186/s13756-019-0645-9

**Published:** 2019-11-28

**Authors:** Vu Thi Ngoc Bich, Le Viet Thanh, Pham Duy Thai, Tran Thi Van Phuong, Melissa Oomen, Christel Driessen, Erik Beuken, Tran Huy Hoang, H. Rogier van Doorn, John Penders, Heiman F. L. Wertheim

**Affiliations:** 1 Oxford University Clinical Research Unit and Welcome Trust Major Asia Programme, Oxford, Vietnam; 2 Department of Medical Microbiology and Radboudumc Center for Infectious Diseases, Radboud University Medical Center, Nijmegen, the Netherlands; 3 Quadram Institute Bioscience, Norwich Research Park, Norwich, UK; 4 National Institute of Hygiene and Epidemiology, Ha Noi, Vietnam; 5 Department of Medical Microbiology, School for Nutrition and Translational Research in Metabolism (NUTRIM) and Care and Public Health Research Institute (Caphri), Maastricht University Medical Center, Maastricht, the Netherlands; 6 Center for Tropical Medicine, Nuffield Department of Clinical Medicine, University of Oxford, Oxford, UK

**Keywords:** Resistome, Vietnam, Environmental resistome, Antibiotic use, *Mcr-1*, *Mcr-3*

## Abstract

**Background:**

Antibiotic resistance is a major global public health threat. Antibiotic use can directly impact the antibiotic resistant genes (ARGs) profile of the human intestinal microbiome and consequently the environment through shedding.

**Methods:**

We determined the resistome of human feces, animal stools, human food and environmental (rain, well, and irrigative water) samples (*n* = 304) in 40 households within a community cohort and related the data to antibiotic consumption. Metagenomic DNA was isolated and qPCR was used to determine presence of mobile colistin resistance (*mcr*) genes, genes encoding extended-spectrum β-lactamases (ESBL), carbapenemases and quinolone resistance genes.

**Results:**

Nearly 40 % (39.5%, 120/304) of samples contained ESBL genes (most frequent were *CTX-M-9* (23.7% [72/304]), *CTX-M-1* (18.8% [57/304]). Quinolone resistance genes (*qnrS*) were detected in all human and 91% (41/45) of animal stool samples. *Mcr-1 and mcr-3* were predominantly detected in human feces at 88% (82/93) and 55% (51/93) and animal feces at 93% (42/45) and 51% (23/45), respectively. *Mcr-2, mrc-4 and mcr-5* were not detected in human feces, and only sporadically (< 6%) in other samples. Carbapenemase-encoding genes were most common in water (15% [14/91]) and cooked food (13% [10/75]) samples, while their prevalence in human and animal stools was lower at 4% in both human (4/93) and animal (2/45) samples. We did not find an association between recent antibiotic consumption and ARGs in human stools. Principal component analysis showed that the resistome differs between ecosystems with a strong separation of ARGs profiles of human and animal stools on the one hand versus cooked food and water samples on the other.

**Conclusions:**

Our study indicated that ARGs were abundant in human and animal stools in a rural Vietnamese community, including ARGs targeting last resort antibiotics. The resistomes of animal and human stools were similar as opposed to the resistomes from water and food sources. No association between antibiotic use and ARG profiles was found in a setting of high background rates of AMR.

## Introduction

Antimicrobial resistance (AMR) has become omnipresent and is still increasing globally, making treatment of infectious diseases more and more challenging [1]. Current research includes assessing and analyzing the resistome: the combined collection of antibiotic resistance conferring genes and their precursors in both pathogenic and non-pathogenic bacteria [2].

AMR is recognized as a public health issue by the Vietnamese Ministry of Health [3]. Despite the introduction of a prescription law in Vietnam in 2005, 88 to 91% of all antibiotics were still sold without prescription in rural and urban private pharmacies in 2014 [4]. Use of antibiotics without prescription is usually inappropriate and considered an important driver of AMR [5]. In Vietnam, resistance to first-line drugs of treatment is high among common bacterial pathogens in both hospital and community settings [4, 6].

Besides human antibiotic consumption, antibiotics are widely used in livestock breeding and aquaculture for disease treatment, prevention and growth promotion [7]. Vietnam’s agricultural sector contributes 30% to the national Gross Domestic Product (GDP) and Vietnam is the 4th largest aquaculture producer globally [8]. Nearly half (43.7%) of commercial feed products contain at least one antibiotic. 66.7% of industrial-scale farms and 20% of household farms used antibiotics as growth promoters for pigs [9]. Since 2017 the use of antibiotics for growth promotion is illegal but the effect of this ban has yet to be determined [10]. This situation is largely responsible for the high proportion of antibiotic-resistant bacteria in food products and aquatic environments [7, 9, 11].

Antibiotic use has an important impact on the intestinal microbiota composition of humans and animals as well as on their surrounding environment. Recent studies considered the gut microbiome as an important antimicrobial resistance gene (ARG) reservoir in which ARGs between native and transient intestinal bacteria can be transferred [12, 13]. In a recent study, a total of 1093 ARGs were identified in 162 fecal metagenomes from human individuals from China, Denmark and Spain [14]. Another study on healthy individuals from seven countries detected ARGs for 50 of 68 classes of antibiotics in 252 fecal metagenomes, at an average of 21 ARGs per sample [15]. Exposure to antibiotic-resistant bacteria or genes present in animal stools, food products and water in combination with antibiotic use may lead to acquisition of ARGs in the human intestinal microbiome [16]. Here, we present longitudinally collected human antibiotic usage data and single time point ARG profiles of human, animal, food and environmental samples in a rural Vietnamese community.

## Methods

### Study population & design

From November 2014 to June 2015, we conducted a longitudinal study examining the impact of antibiotic use on the microbiome and resistome of humans, their food, animals and the environment in Vietnam. The population of this study was selected from an existing prospective, household-based community cohort established to investigate influenza virus transmission in the commune of Thanh Ha in Ha Nam province, Vietnam in 2007 [17]. From this community, we selected 80 households: 40 households with children under 11 years of age and 40 households without children under 11 years of age. At baseline, a semi-structured questionnaire was used to record demographic characteristics, social economic factors, living conditions, health status. Current antibiotic use data was collected weekly. In case of antibiotic use, we collected the information of drug name, dose, supplier, and indication for drug use.

Household members were included if they were willing to participate and were available for follow up. Household members were excluded if they were unable to collect fecal specimens, were known to be on antibiotic maintenance therapy or had a chronic intestinal condition.

Participants received a clean container with spoon attached to the lid (VWR corporate Headquaters, PA, USA), gloves, and a biohazard bag and study staff demonstrated the for home collection of the stool sample [18]. Processed food and water samples were collected following the sampling protocol for low-biomass samples as described elsewhere [19]. Fresh stool, processed food and water samples with the collection date and time were collected by study staff and sent in cold condition (4–8 °C) to the National Institute of Hygiene and Epidemiology (NIHE) and Oxford University Clinical Research Unit for sample processing within 4 h. Samples were stored at − 80 °C until they were used for microbiome and resistome analysis in Maastricht, Netherlands.

**Fig. 1:**
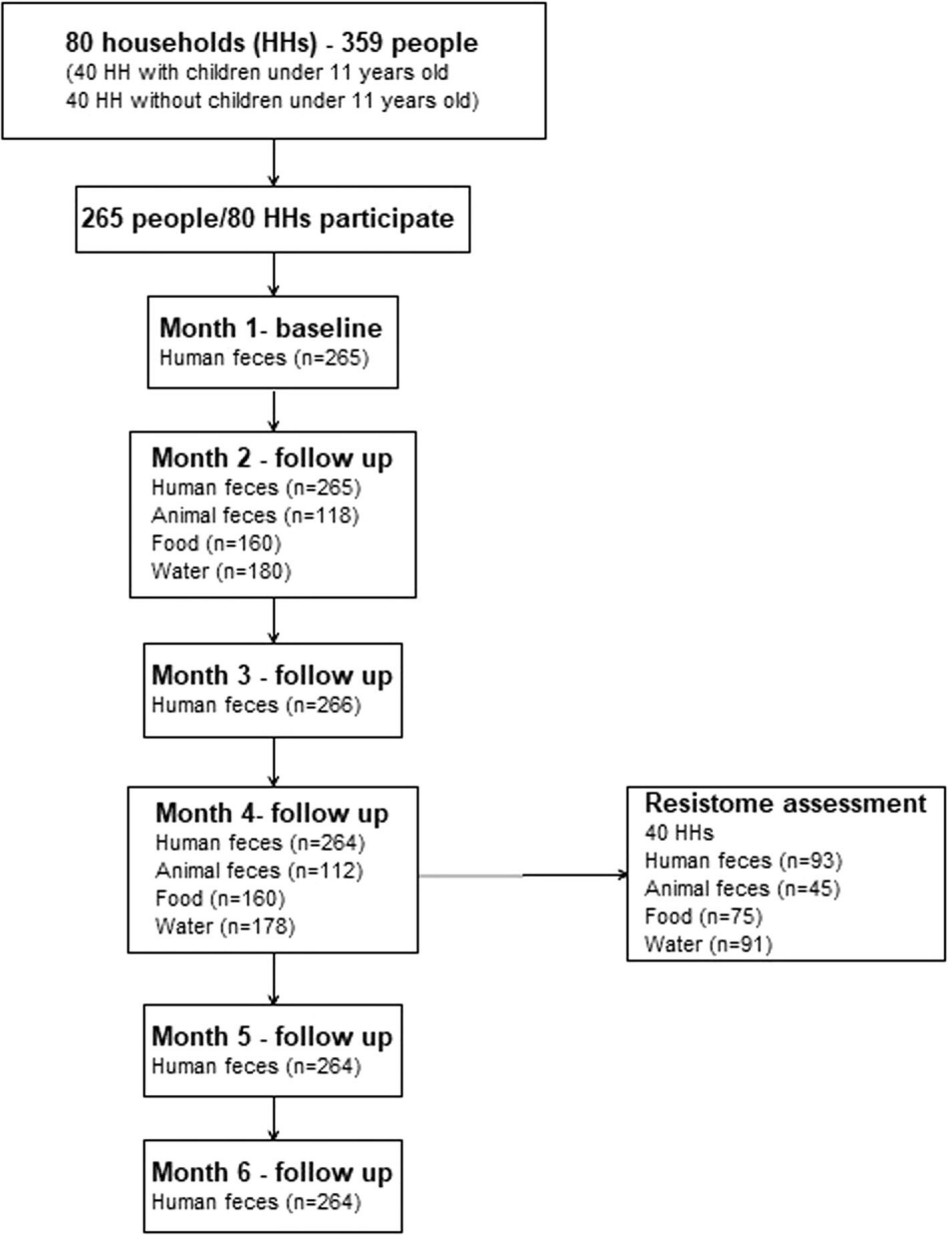
Sample population of study and sample selected of ARG explorative study

For the purpose of analyzing the resistome in this study, a subset of 40 out of the 80 households was selected: 20 households in which at least one member used any beta-lactam antibiotic and 20 households in which no antibiotic use was reported prior to sampling. At 4 months, we collected domestic animal feces, cooked food products and water samples from these households (Fig. 1). Three hundred four samples were collected including 93 human fecal samples, 45 animal fecal samples, 75 cooked food samples and 91 water samples (Fig. 1).

The research was approved by the Oxford University Tropical Research Ethics Committee (OxTREC, 49–14) and the National Institute of Hygiene and Epidemiology, Vietnam (NIHE) institutional review board. Written informed consent was obtained from all participants in the study.

### DNA extraction

Repeated Bead-Beating (RBB) combined with column-based purification was used to extract DNA from human and animal fecal samples according to protocol Q (IHMS_SOP 06 V2 - http://www.microbiome-standards.org/index.php?id=253) of the International Human Microbiome Standards consortium [20]. Bead-beating was done using the FastPrep™ Instrument (MP Biomedicals, Santa Ana (CA), USA) with 0.1 mm zirconium-silica beads (BioSpec Products, Bartlesville (OK), USA) to homogenize feces. DNA was finally purified by adapting to QIAamp DNA Stool Mini kit columns (Qiagen, Hilden, Germany).

Regarding the isolation of metagenomic DNA from processed food, 200 mg of sample was homogenized in 0.75 ml PBS (pH 7.2), and centrifuged for 3 min at 13,000 x g. Metagenomic DNA was subsequently isolated by the QIAGEN DNeasy Power Food Kit according to the manufacturer’s instructions for DNA isolation from solid food. For isolation of microbial DNA from water samples, 100 ml of collected water was filtered through 0.22 μm mixed cellulose esters membrane filters (Sartorius, Göttingen, Germany) to capture bacteria. One quarter of the filters were used for metagenomic DNA extraction using the QIAGEN DNeasy Power Water kit according to the manufacturer’s protocol.

Upon isolation, DNA concentrations were determined using the Quan-iT PicoGreen dsDNA assay (Invitrogen, Carlsbad (CA), USA).

### Quantitative detection of ARG identification and quantification by qPCR

qPCR amplification was performed to quantify the total number of 16S ribosomal RNA gene copies (16S rDNA) using MyiQ single – color real time PCR detection system (Bio-Rad, Hercules (CA), USA) as previously described [21] (Additonal file 1: Supplement 1).

The selected samples were also assessed for the presence and abundance of ARGs by quantitative real-time PCRs using a 7900HT Fast Real-time PCR System (Applied Biosystem Inc., Foster City (CA), USA) (Additonal file 1: Supplement 2).

The qPCRs to detect and to quantify the ESBL, carbapenemases and quinolone resistance genes were performed using primers and probes from previous studies [22–25] and subsequently validated protocols for their application on metagenomics DNA [26–28] (Additonal file 1: Supplement 1).

In order to identify and quantify plasmid-mediated colistin resistance (*mcr*) genes (*mcr-1*, *mcr-2, mcr-3*, *mcr-4*, *mcr-5*), we used in-house designed procedures, specifically for metagenomics DNA (Additional file 1: Supplement 1), from the Department of Medical Microbiology at the Maastricht University Medical Center. For all mcr-genes, products from positive samples were sequenced by using the PCR primers and an ABI BigDye Termination v1.1 Cycle Sequencing Kit (Applied Biosystems, Foster City, CA, USA). Sequencing data were analyzed by using BLAST (http://blast.ncbi.nlm.nih.gov/Blast.cgiExternal Link).

Quantification of the abundance of AMR genes in samples was achieved by comparing the cycle threshold (Ct) values to a standard curve. The standard curses were constructed by subjecting serial 10-fold dilutions of positive plasmid constructs containing the AMR target sequences to the same qPCR assays.

The load of AMR genes in samples was determined using standard curves generated by serial dilutions of plasmid constructs containing the AMR gene of interest. Subsequently, we calculated the ratio between AMR gene copies and 16S rRNA gene copies to adjust for differences in bacterial load between samples. Samples that were negative for a particular ARG, were assigned a ARG copy number of zero.

### Statistical analysis

Pearson’s chi-square test, or Fisher’s exact test in case of expected cell numbers < 5, was used when comparing the prevalence of ARGs or other dichotomous data between two groups. The statistical significance level was set at 5%.

To examine the association between the relative abundance of resistance genes in fecal samples and human antibiotic consumption, the ratio of ARGs to 16S rRNA gene copy numbers was calculated. The differential abundance of ARGs among the two antibiotic exposure groups was compared by Wilcoxon test (IBM SPSS Statistics v23.0, IBM, Armonk (NY), USA).

We applied principal component analysis (PCA) from the stats package and used ggbiplot package in R 3.5.3 to visualize the ordination of the ARG profiles generated from human and animal fecal samples, cooked food products and water. Before running PCA, we standardized the data to equally scale all variables and take into account zeros (samples without a specific AMR gene) in the data set.

## Results

### Demographics of the complete cohort

At baseline, demographic information from 359 individuals from the selected 80 households in the ongoing Ha Nam household cohort study on influenza was available: 189 (52.6%) individuals were female, 192 (53.5%) individuals belonged to the working age group (19 to 55 years of age), and 61 (17.0%) were children under 11 years of age. With 154 (42.9%) farmers, farming was the most common occupation. Thirty-one (8.6%) of the participants had an underlying chronic illness. Two hundred sixty-fivepeople were willing to participate in this study, 94 of 359 people were not willing or unable to participate in this study. The distribution of demographic characteristics of participants was comparable to that of the total population (Table 1a).

**Table 1:** Study population characteristics (1.a) Age distribution occupation and antibiotic use, (1.b) direct environmental characteristics of households

1.a			
Variables	No. (%)		
	Resident (n = 359)	Enrolled (n = 265)	Resistome analysis (n = 93)
*Age group (years) (Mean + SD)*	32.1 ± 19.2	32.4 ± 19.6	29.4 ± 18.9
0 to 11	61 (17.0)	54 (20.4)	23 (25)
12 to 18	52 (14.5)	39 (14.7)	12 (13)
19 to 55	192 (53.5)	133 (50.2)	50 (54)
> 56	54 (15.0)	39 (14.7)	8 (9.0)
*Male sex*	170 (47.4)	117 (44.5)	38 (41)
*Occupation*			
Farmer	154 (42.9)	115 (43.0)	36 (39)
Health care staff	6 (1.7)	2 (0.8)	0 (0)
Worker	36 (10.0)	23 (8.7)	10 (11)
Others (teacher, engineer, accountant, etc)	47 (13.1)	28 (10.6)	13 (11)
Antibiotic use during 6 months follow up			
Yes	97 (27.0)	93 (35.1)	39 (42)
Beta lactam (amoxicillin, cefepime, cefexime, cephalexin, penicillin)	88 (90.7)	84 (90.3)	39 (100)
1.b			
Variables	No. (%)		
	Number of HH (n = 80)		HHs in resistome analysis (n = 40)
Main water			
Rain water	75 (94)		38 (95)
Drain well water	74 (93)		36 (90)
Cleaned water	5 (6)		2 (5)
River water	16 (20)		6 (15)
Domestic animal			
Chicken	56 (70)		27 (68)
Dog	63 (79)		31 (78)
Pig	9 (11)		5 (12)
Other (cow, duck, goose, bird, etc)	62 (78)		
Origin of fertilizer used for vegetable culture			
Human	1 (1)		1 (2)
Animal	13 (16)		8 (20)
Chemical	27 (34)		14 (35)

All 80 households had at least one animal, with dogs in 63 (79%) of the households and chickens in 56 (70%) being most common. In 62 (70%) households, at least one animal species other than dogs or chicken was present, mostly pigs and cows. Rainwater was the main water supply for 75 (94%) households for cooking and well water was the main water source in 74 (93%) households for daily activities such as washing clothes, vegetable, raw meat. We observed that 38 (48%) households used fertilizer in vegetable culture, of which 14 households (37%) used fertilizer of human or animal fecal origin (Table 1b).

### Antibiotic consumption in the complete cohort during 6 months of the study follow-up

During the 6 months follow-up period, 97 (27.0%) participants belonging to 37 households used antibiotics at least once. 55 (56.7%) of them reported use of a single course of antibiotics, 23 (23.7%) used two courses of antibiotics and 18 (18.6%) individuals used more than two courses during follow-up. Antibiotics were obtained from various sources in the drug supply system: national level referral hospitals (3%, *n* = 4), provincial level hospital (8%, *n* = 15), community level healthcare center (42%, *n* = 74), private pharmacy (40%, *n* = 71) or through antibiotic-sharing (8%, *n* = 14). Beta-lactam antibiotics were most commonly used: 79 (82%) of 96 individuals who consumed antibiotic, had at least one course of beta-lactam antibiotics (1st generation cephalosporin [43%], amoxicillin [22%], 2nd or 3rd generation cephalosporin [17%], penicillin [9%]).

### Resistome of a subset of cohort at the fourth month follow-up time-point

A total of 304 human, animal, food and environmental samples were collected at month 4 from 40 households (see above). Samples were subjected to qPCR assays to assess the presence and abundance of ARGs. A total of 14 unique ARGs conferring resistance to different classes of antimicrobials were targeted. The number of detected resistance genes per individual sample ranged between 1 and 8. *QnrS* dominated in human, animal and environmental samples (220/304, 72.4%), followed by *mcr*-1 (134/304, 44.1%), *CTX-M* (127/304, 41.8%) and *mcr*-3 (91/304, 29.9%). Other commonly detected genes were: *qnrA* (39/304, 12.8%), *bla*_*NDM*_ (25/304, 8.2%), *mcr*-2, *mcr*-4, *mcr*-5 and *bla*_OXA-48_ were found only sporadically in the various sample types (Table 2). Proportions of ARG positive human and animal samples were significantly higher than of cooked food and water. In particular, *qnrS* was found in all (93/93) of the human fecal samples and in 91% (41/45) of animal stools whereas 75% (57/75) and 32% (29/91) of the cooked food and water samples were positive, respectively. In contrast, the prevalence of *qnrA* was low in all sample types except animal faeces (22/45, 49%). *Mcr*-1 was detected at a high frequency in animal (42/45, 93%) and human stools (82/93, 88%), and at lower rates in cooked food (9/75, 12%) and water (1/91, 1%).

**Table 2:** The prevalence of AGRs among human, animal, food and water obtained from a subset 304 samples from 40 households

Antibiotic resistance genes (ARGs)	Human (n, %)^‡^ *n* = 93	Animal (n, %)^‡^ *n* = 45	Food (n, %)^‡^ *n* = 75	Water (n, %)^‡^ *n* = 91
*mcr*-1	82 (88)^b, c^	42 (93)^d, e^	10 (13)^b, d, f^	2 (2)^c, e, f^
*mcr*-2	0 (0)	0 (0)	1 (1)	0 (0)
*mcr*-3	51 (55)^b, c^	23 (51)^d, e^	21 (28)^b, d, f^	6 (7)^c, e, f^
*mcr*-4	0 (0)	1 (2)	1 (1)	0 (0)
*mcr*-5	0 (0)	2 (4)	3 (4)	5 (6)
*bla*_NDM,_	4 (4)^b^	2 (4)	10 (13)^b^	14 (15)
*bla*_KPC_	0 (0)	0 (0)	0 (0)	0 (0)
*bla*_OXA-48_	0 (0)^b^	0 (0)	6 (8)^b, f^	2 (2)^f^
*bla*_VIM_	0 (0)	0 (0)	0 (0)	0 (0)
*CTX-M-1*	35 (38)^b, c^	15 (33)^d, e^	5 (7)^b, d^	2 (2)^c, e^
*CTX-M-2*	17 (18)^c^	6 (13)^e^	17 (22)^f^	0 (0)^c, f^
*CTXM-9*	57 (61)^a,b,c^	13 (29)^a,d,e^	2 (3)^b, d^	0 (0)^c, e^
*qnrA*	9 (9)^a^	22 (49)^a,d,e^	9 (12)^d, f^	5 (6)^e, f^
*qnrS*	93 (100)^a,b,c^	41 (91)^a,d,e^	59 (78)^b, d, f^	30 (33)^c,e,f^

^‡^Differential proportion of ARGs among sample types was compared using Pearson’s Chi square or Fisher’s exact. Letters a,b,c,d,e,f indicate statistically significant differences (*P*-value< 0.05) between two sample types (^a^human animal, ^b^human and food, ^c^human and water, ^d^animal and food, ^e^animal and water, ^f^water and food)

*Mcr-3* was found in 55% (51/93) of human stools followed by 51% (23/45) in animal stools, 28% (21/75) in cooked food and 7% (6/91) in water samples. Human stools also frequently contained the following ESBL-encoding genes: *CTX-M-1* (38%, 35/93), *CTX-M-2*(18%, 17/93) and *CTX-M-9* (61%, 57/93). The proportion of *CTX-M-1* and *CTX-M*-2 (13%, 6/45) positive animal stools was similar to the positive proportion of human samples, whereas the proportion positive of *CTX-M*-9 was much lower (*p* < 0.001) in animal stools (29%, 13/45). The proportion of *CTX-M-1*, *CTX-M-2* and *CTX-M-9* groups in cooked food and water were much lower (*p* < 0.001) than in human and animal stools (Table 2).

As opposed to the other ARGs, the proportion positive for carbapenemase encoding genes was highest in food and water samples. The carbapenemase encoding gene *bla*_NDM_ was detected in 13% (10/75) of food and 15% (14/91) of water samples, but only in 4% (4/93) of human and 4% (2/45) of animal stools. Moreover, while bla_OXA-48_ was absent in human and animal samples, 8% (6/75) of food and 2% (2/91) of water samples were positive for this carbapenemase gene. *Bla*_KPC_ and *bla*_VIM_ were not found in any of the samples (Table 2).

### ARG in relation to antibiotic consumption in humans

During the 4 months prior to sampling, 44 (47%) of 93 participants included for the resistome analysis took antibiotics. Table 3 shows the proportion of fecal samples from participants who did and did not use antibiotics positive for specific ARGs. The proportion of *qnrA* was significantly lower (1/49 versus 7/44) among individuals that consumed antibiotics in the 4 months prior to sample collection (Fisher exact, *p* –value < 0.05). The proportion of the remaining ARG did not differ significantly between the two groups (Table 3).

**Table 3:** The proportion of ARGs in the comparison between humans who used antibiotic and those who did not use antibiotics

Antibiotic resistance genes (ARGs)	No use antibiotic, n/N (%)	Use antibiotic, n/N (%)	*p* -value^‡^
*qnrA*	1/49 (2)	7/44 (16)	0.025
*qnrS*	49/49 (100)	44/44 (100)	1
*CTX-M-1*	14/49 (29)	21/44 (48)	0.086
*CTX-M-2*	8/49 (16)	9/44 (20)	0.789
*CTX-M-9*	29/49 (59)	28/44 (64)	0.676
*mcr-1*	44/49 (90)	38/44 (86)	0.751
*mcr-3*	23/49 (47)	24/44 (54)	0.535
*bla*_NDM_	2/49 (4)	2/44 (5)	1

^‡^Differential proportion of ARGs among two groups was compared using Pearson’s Chi square or Fisher’s exact, *P*-value < 0.05 was considered significant

**Fig. 2:**
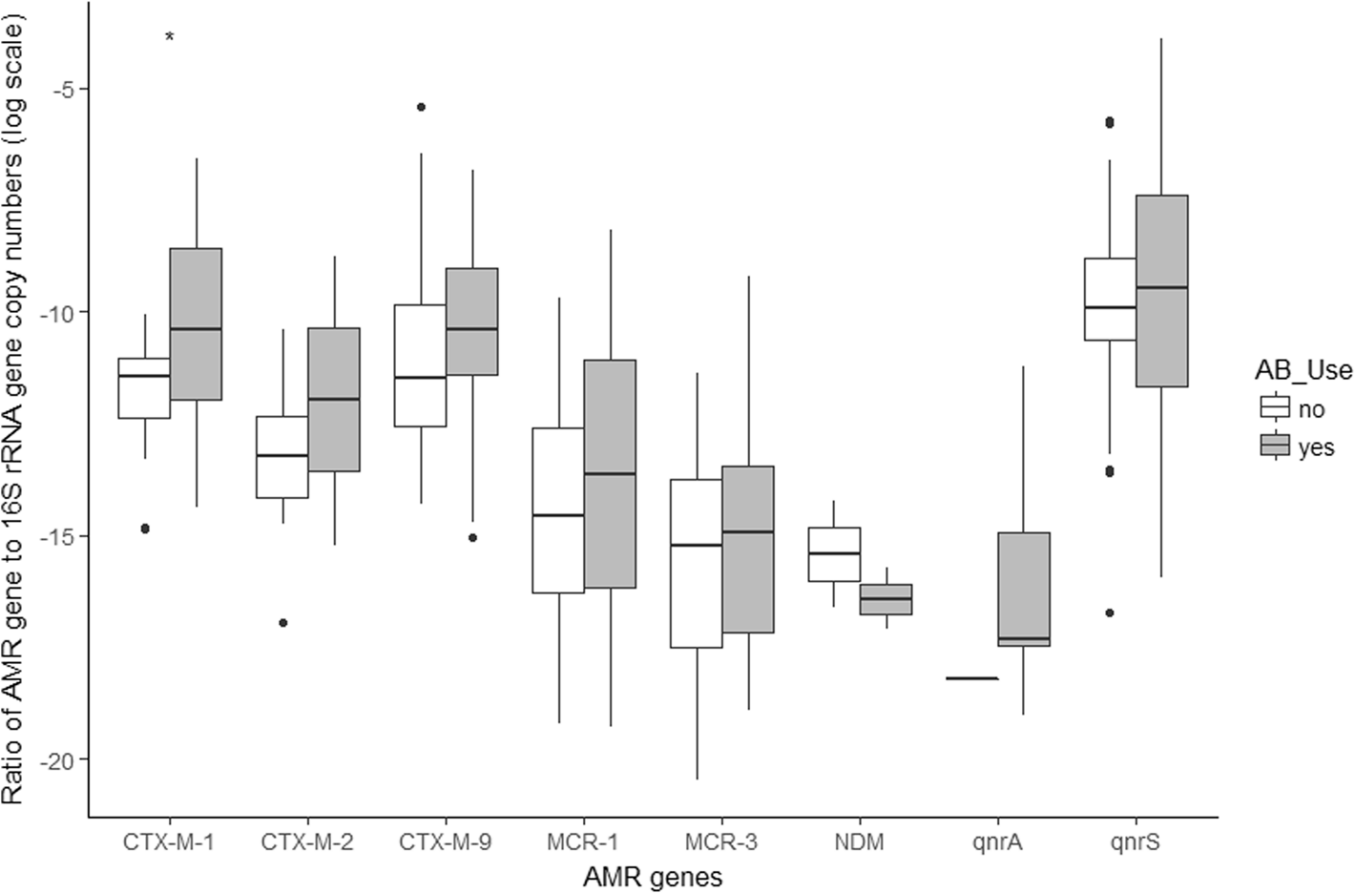
Comparison of the relative abundance of ARGs between people who used antibiotics versus those who did not use antibiotics in the 4 months preceding sample collection. *indicates *p* < 0.05 as determined by Wilcoxon test

In terms of ARG abundance in fecal samples from participants who used versus those who did not use antibiotics**,** we only observed a statistically significant higher relative abundance of the *CTX-M-1* group among participants who used antibiotics (*p* < 0.05). For the remaining ARGs no statistically significant differences were found (Fig. 2).

**Fig. 3:**
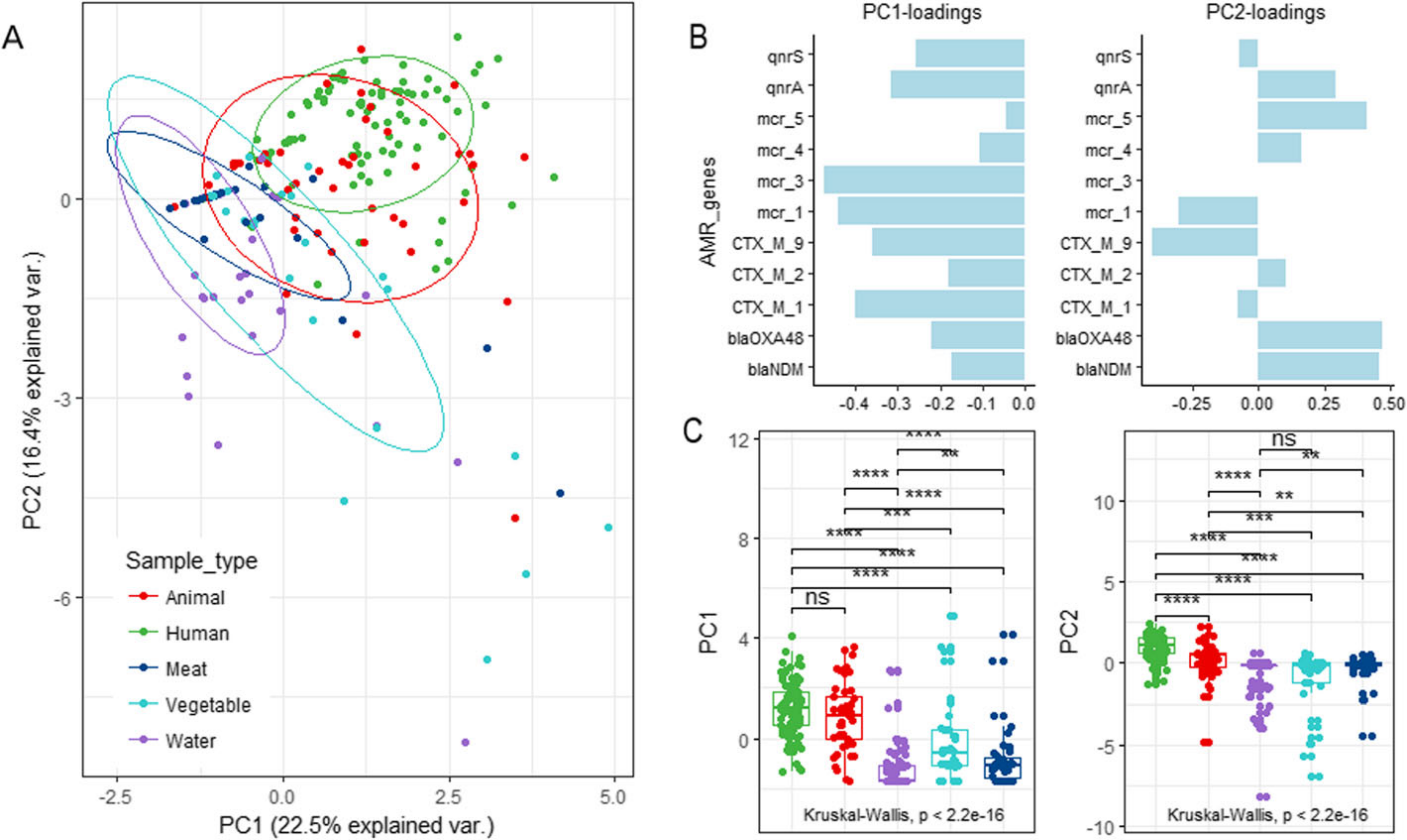
Principal Component Analysis (PCA) plots (**a**) showing the ordination of samples according to their ARGs profiles. Samples are colored according to their origin: animal feces (red), human feces (green), meat (dark blue), vegetable (light blue) or water (purple). The ellipses represent a 95% confidence interval of samples of the same origin. Loading scores (**b**) of the ARGs on the first and second principal component. Boxplots (**c**) showing the scores on the first and second principle component of samples of the same origin

We subsequently conducted a principal component analysis (PCA) to visualize the ordination of ARGs abundance between sample types. ARGs profiles of human and animal stools appeared to be most strongly separated from the other sample types in both the first two principal components (PC1 and PC2) which accounted for the first and the second largest possible variance in our data set (Fig. 3a). Testing for potential differences confirmed that scores on PC1 significantly differed between all sample types, except for human and animal samples. Also, the scores on PC2 are significantly different between most of the sample types, except for water and vegetables, indicating that the resistome differs between sample types (Fig. 3c). In order to identify how the sample types differed in resistome composition, we next plotted the factor loadings (the correlation coefficients between the ARGs abundance and sample types) of the PC1 and PC2. The plot shows that the abundance of *mcr-1*, *mcr-3* and *CTX-M-1* genes are highly loaded on PC1 and abundance of *bla*_*NDM*_, *bla*_*OXA-48*_, and *mcr-5* genes dominated on PC2 (Fig. 3b). This revealed that *mcr*-1, *mcr*-3, and *CTX-M-1* variations are mainly driving the separation of the different sample types on PC1, whereas *bla*_*NDM*_ *bla*_*OXA-48*_, and *mcr-5* are contributing to the separation on PC2 (Fig. 3b).

## Discussion

We used a targeted metagenomics approach to explore the resistome of humans and animals and their environment in northern Vietnam. Our study revealed highly variable detection rates between sample types, indicating that resistome profiles are habitat-dependent.

The detection of *mcr-1* in both human (88%, 82/93) and animal (93%, 42/45) stools was higher than in other parts of the world. Using similar metagenomic screening, the acquisition rate of *mcr-1* was approximately 4.9% (6/122) among Dutch intercontinental travelers, while none of the participants carried *mcr-1* prior to travel departure [26]. A culture-based screening failed to detect *mcr-1* positive *Enterobacteriaceae* in healthy Swiss individuals [4]. Although previous studies in Asian populations also showed high *mcr-1* detection rates, proportions were still much lower than in our study (ranging from 0.4 to 2%). A recent study conducted in southern Vietnam reported *mcr-1* carriage among 21% of humans and 59% of chickens [29]. The latter study reported highest human *mcr-1* colonization rates among farmers exposed to *mcr-1* positive chickens, suggesting zoonotic transmission. A study by Chen et al., that investigated the presence of *mcr-1* in the ecosystem in China from 2015 to 2016 showed 51% (234/480) of animal faeces were *mcr-1* positive [30]. Interestingly, this study reported much higher *mcr-1* rates in water (71%, 24/34) and food (36%, 486/1371) than our findings [30].

The high *mcr-1* detection rates in our study when compared to previous studies conducted in Vietnam and China is most likely due to widespread use of colistin in animal feed but could also partly be attributed to the culture-independent targeted metagenomic approach, which is known to have a higher sensitivity when compared to culture-based approaches, and also detects ARGs from non-viable bacteria and extracellular DNA. Using our approach provides better insight on colistin resistance gene abundance in a sample type rather than a culture based approach of indicator bacteria.

The *mcr-3* gene is evolutionarily distinct from *mcr-1* [31] and both have successfully spread among *Enterobacteriaceae* in Vietnam, as previous studies indicated [29, 32]. Studies about human fecal shedding of *mcr-3* in the community are limited. Here, the detection rate of *mcr-3* (55%, 51/93) in human feces was much higher than reported previously (5% [8/152] from stool samples from outpatients in China using culture based approaches) [33].

Examining the overall resistome profiles indicated that the separation of human and animal fecal samples from the environmental samples was mainly driven by *mcr-1* and *mcr-3*. There is limited evidence of ESBLs transmission from animals and meat to humans [34–36], but our results suggest its likely that *mcr* ARGs are transferred from animals to humans as colistin is only used in animal husbandry [7, 37]. Altogether our results advocate a comprehensive assessment of the acquisition and subsequent transmission of *mcr* genes between animals and humans.

The detection rate of *bla*_NDM_ in human feces was relatively low, and comparable to the prevalence of Gram-negative bacilli carrying *bla*_NDM_ cultured from throat swab in humans from rural northern Vietnam [38]. However, it is interesting to note that *bla*_NDM_ and bla_OXA-48_ were more frequently detected in well and irrigative water and food than in human and animal feces. Possibly, carbapenemase genes in environmental bacteria as *Acinetobacter spp.* are much more common than in human and animal intestinal bacteria such as *E. coli* and this may be a result of higher abundance of these bacteria in aquatic environments. Possibly, residues of antibiotics and other resistance gene driving pollutants (e.g. heavy metals) may be present in this environment selecting for resistance [39].

Antibiotic use in humans and animals in Vietnam is largely uncontrolled [40, 41] as reflected by the findings from our cohort where beta-lactams (mostly cephalosporins) and other antibiotics were very commonly used. However, we only found a slightly higher richness of *CTX-M-1* genes, and no differences for other genes, among individuals with recent use of beta-lactam antibiotics. The long-term frequent exposure to antibiotics may have led to a ‘stable’ omnipresence of ARGs. Alternatively, increases in the number of ESBL producing *E.coli* and *Salmonella spp* also occur without any prior use of cephalosporins [42], potentially explaining the lack of association between recent antibiotic use and the presence and abundance of most ARGs in our study.

The present study was mainly explorative and has several limitations. Because of the cross-sectional nature of the analyzed sample collection it is impossible to study the transmission dynamics of ARGs, as well as the direct impact of antibiotic use on the ARG profile. Moreover, compared with culturing methods, a metagenomic approach has the advantage of being able to detect resistance in a much wider array of species; however, we did not assess in which organisms the ARG detected in our study were present, nor if they were being expressed.

The targeted metagenomics approach also provides higher sensitivity to detect ARGs than culture-based methods and sequence-based metagenomics [43, 44]. Using sequence-based metagenomics, a recent study monitored antimicrobial resistance in urban sewage from 60 countries [45]. Although this study showed that *mcr-1* was detected in only a few countries, including Vietnam, the low relative abundance of *mcr-1* and absence of *mcr-3* in Vietnamese sewage underestimated the wide dissemination of *mcr* genes in the Vietnamese community as we describe here. This finding indicates that targeted metagenomics can complement sequence-based metagenomics screening to monitor antimicrobial resistance within local communities and across the globe.

In order to acquire a more detailed insight into the impact of antibiotic use on resistome profiles and to understand the dynamics of the resistome in various microbial ecosystems, longitudinal analyses are needed. These analyses are underway using samples from our cohort collected at different time points. Moreover, additional profiling of the microbiome composition will enable more accurate analysis of the correlation between resistome and taxonomic changes in response to antibiotics in a high ARG and usage background.

## Conclusion

Our study provides a comprehensive view of ARGs in humans, animals, and their environment in rural northern Vietnam and reveals a high level of circulating ARGs with different resistome profiles per sample type. Mobilized colistin resistance genes (m*cr-1 and mcr-3)* were detected in human feces at 88% (82/93) and 55% (51/93) and animal feces at 93% (42/45) and 51% (23/45), respectively. Carbapenemase genes in the environment are much more common than in human and animal feces. Although beta-lactam antibiotics were used most frequently in our study community, their impact on the presence and abundance of ARGs was limited and needs to be examined in more detail in a longitudinal study.

## Additional file

**Additional file 1: Supplement 1.** List of primers and probes used to identify and quantify the ARGs by qPCR. **Supplement 2.** qPCR's results of ARGs identification and quantification.

## Data Availability

Please contact to Corresponding Author. *email: bichvtn@oucru.org

## Abbreviations

AMR: Antimicrobial resistance
ARGs: Antibiotic resistant genes
ESBL: *Extended spectrum beta-lactamase*
GDP: Gross Domestic Product
Mcr: Mobilized colistin resistance
PC1: Principal component 1
PC2: Principal component 2
PCA: Principal component analysis
